# Attitudes to weight and weight management in the early teenage years: a qualitative study of parental perceptions and views

**DOI:** 10.1111/hex.12182

**Published:** 2014-02-25

**Authors:** Wendy J. Wills, Julia Lawton

**Affiliations:** ^1^ Centre for Research in Primary and Community Care (CRIPACC)University of HertfordshireHatfieldUK; ^2^ Centre for Public Health SciencesUniversity of EdinburghEdinburghUK

**Keywords:** parents, qualitative study, socio‐economic status, weight management, young teenagers

## Abstract

**Background:**

As most young teenagers grow up in families, parents might be well situated to facilitate and support their weight management and thereby prevent or manage obesity prior to adulthood.

**Aim:**

This paper explores parents' perceptions of, and views about, their teenage children's weight and the factors that influence parents' weight management strategies.

**Design, setting and participants:**

We conducted two qualitative studies in Scotland, UK, involving in‐depth interviews with the parents of overweight/obese and ‘normal’ weight 13–15 year olds (*n* = 69).

**Findings:**

Parents' concerns about their own weight provided useful context for understanding their attitudes or actions with regards to their teenage child. Some parents described their teenager's weight as being of concern to them, although puberty often introduced confusion about a child's weight status. Genetic explanations were very often put forward as a way of making sense of teenage weight or body size. Frustration about advising teenagers about weight management was expressed, and some parents worried about giving their growing child a ‘problem’ if they directly raised concerns about weight with them.

**Discussion:**

Parents' views about their own weight as well as social and moral norms about labelling a teenager as overweight or as needing help with their weight could usefully inform patient‐centred service development. Parent/teenage partnerships and supporting parents to create a healthy home in which teenagers can make healthier choices are suggestions for intervention development.

**Conclusion:**

The study highlights the importance of taking parents' perceptions into account when developing family‐based interventions to address teenage overweight and obesity.

## Introduction

As most teenagers grow up in families, parents might be well situated to facilitate and support their weight management and thereby prevent or manage obesity prior to adulthood. This paper explores how parents perceive weight in adolescence and their attitudes towards, and experiences of, promoting weight management at this point in their child's life course. It draws upon two qualitative studies involving parents of young teenagers (aged 13–15 years), from different socio‐economic groups living in Scotland, UK.

Children who have one, or two, obese parents are more likely to be obese than children whose parents are not overweight or obese.^1^ There is also strong evidence to suggest that obesity is related to socio‐economic status (SES), with families from lower SES groups having a higher prevalence of obesity than other groups.^2^ Research also suggests that adults from high SES groups are more likely to value and prioritise ‘body maintenance’ and to express a desire to control and shape their own health and wellbeing.^3^, ^4^ People from lower SES groups also have concerns about weight and health, but, for these individuals, other aspects of daily life often take precedence, like concerns about employment, their neighbourhood and their teenager's safety.^5^ Such prioritizing is informed by social and cultural values which are embedded through living everyday life in close proximity with ‘like minded’ people.^5^ As parents have an important role to play in managing health and wellbeing throughout childhood, it is essential to understand the mechanisms and processes through which parents perceive, influence and manage their teenage children's weight status.

## Methods

Two linked qualitative studies were conducted; the first (2002–05) focused on families with young teenagers who had a lower SES and the second (2006–08) involved families with teenagers from higher SES groups. Both studies received University ethics approval and both employed the same research design.

For both studies, young people aged 13–15 years were recruited via schools in eastern Scotland, which were targeted according to the SES of the student catchment area. The research team made classroom visits to explain what the research involved and, prior to these visits, parents were given the opportunity to withdraw their child from being considered for participation in the research; very few did so.^6^ A small number of young people were also recruited into the first study via youth groups.

All consenting teenagers completed a short screening questionnaire during classroom visits, which asked for socio‐demographic information (e.g. who lived in their household; parent/s' occupations; home address) and about family affluence (e.g. number of holidays taken; whether the family had access to a car and whether the teenager shared a bedroom with a sibling).^7^ Consenting teenagers also had their weight and height measured to calculate their Body Mass Index (BMI). Participants were classified as having a ‘normal’ or ‘overweight/obese’ BMI according to standard cut‐off thresholds.^8^

Information from the screening questionnaires was used to classify families as ‘lower’ or ‘higher’ SES. Young people from more deprived neighbourhoods, who had parent/s without jobs or who worked in manual/unskilled occupations, were classified as being of lower SES, whereas young people from the least deprived neighbourhoods, who had parent/s in skilled/professional occupations, were classified as being of higher SES. The information about family affluence was used as a way of classifying individuals within these higher and lower SES categories.

In the first study, 36 lower SES teenagers were selected, split equally by gender and weight status (half were overweight/obese and half were within the normal range BMI for their gender and age). Thirty six higher SES participants were selected for the second study using the same criteria. Individual interviews were conducted with each consenting teenager in the home setting and findings from these interviews have been reported.^5^, ^9^, ^10^, ^11^ The parent or guardian nominated by each young person as being the family member responsible for the majority of food preparation at home was then recruited. Sixty nine parents/guardians were interviewed across the two studies, including 60 mothers, three fathers and six grandparents. The adult participants are referred to as ‘parents’ in the paper unless making explicit reference to differences between mothers and fathers. Parents were not informed of their child's BMI status and data collected during the teenage interview was not disclosed to parents.

The parent topic guide covered the family's daily food habits (including what, where and when individuals within the family ate) and parents' views about weight, body size, experiences of weight loss and general health in relation to the participating teenager, other family members and other people they knew. Interviews lasted approximately 1 h, were digitally recorded (with consent) and transcribed verbatim.

In both studies, data collection and analysis took place concurrently allowing issues and themes identified in early interviews to inform areas explored in later ones, in line with a grounded theory approach.^12^ Transcripts were read repeatedly by team members to identify key themes.^13^ Fieldwork and analysis conducted for the second study highlighted some new themes and findings; therefore, we revisited the data collected and analysed during the first study. These comparative analyses were undertaken systematically and rigorously across both datasets to verify whether themes were evident, within and across SES groups.^14^, ^15^ We repeatedly checked the data for disconfirming evidence, and disagreements about themes between members of the research team were constantly revisited until consensus about coding was achieved. Pseudonyms are used throughout this paper. As we did not find any striking differences according to SES, findings from across the two studies are presented together.

## Findings

### Parents' experiences of managing their own weight

We were primarily interested in parent's perceptions of, and views about, their children's weight, and this was clearly explained in the study information sheet. However, when asked about these issues, almost all the parents interviewed spontaneously raised their own weight issues and weight management histories. While some parents did not explicitly link their own weight management experiences with how they viewed or managed their teenager's weight, these findings provide useful context when trying to understand parents' attitudes or actions.

Almost every parent interviewed described their own weight as being problematic, and often their weight gain was perceived as something which had built up over a prolonged period of time. Many parents also described their spouses as overweight. For most participants, weight surveillance was viewed as an on‐going process, a source of underlying irritation and an inevitable aspect of getting older. When their own weight was viewed as problematic, parents put forward a range of strategies for trying to manage it, which were often perceived as having limited or temporary effects, for example:

I've been on a diet for about 12 years now [laughing]…I've tried everything, I've tried all those magic tablets, and they do nothing so I'm going to Weight Watchers just now (Angela's mother; child's BMI category: normal)

Some mothers and grandmothers said that their weight had increased once they had had children or that the situation had escalated once they had approached middle age or the menopause, but others, like Ailsa's mother below, also reported battles with weight that had begun when they were children or teenagers:

I mean, I did it when I was 13, I just starved myself and I don't think it did me an awful lot of good whatsoever (Ailsa's mother; child's BMI category: obese)

In terms of not doing her an ‘awful lot of good’, Ailsa's mother believed that her early crash dieting behaviour had stunted her ‘proper growth’ and had had a particularly detrimental effect, she said, ‘on [her] bust’. She also described this experience as having influenced her approach to managing her daughter's weight loss behaviour:

Yeah, well she, I suppose, went on a diet for a few days and gave up but I mean I still think she's a bit young to go on a diet as such so I think it's better when you're not fully growing, it can affect your bust, your general shape so I think just be sensible (Ailsa's mother; child's BMI category: obese)

### Parents' perceptions of teenagers' weight and body size

Parents of some normal and overweight BMI girls and boys expressed the view that their child had developed a ‘tummy’ or ‘stomach’, which seemed to be one way that parents could report that their child was carrying some ‘extra’ weight. This was either viewed as acceptable, or, sometimes, parents reported uncertainty but erred on the side of finding this weight or body shape acceptable. Many parents fell into this category, which highlights the complexities and difficulties of deciding whether to classify one's own child as ‘normal’ or overweight. Puberty also introduced confusion about a child's weight status, with menarche and changes in body shape raising concerns for some parents, as they were unsure if weight gain, or what level of weight gain, was ‘normal’ during this time in their child's physical development:

He went through a stage he was… wasn't fat but he was, he, he had a lot more on him than he does now, now he's much leaner and more muscular. I think they do when they, they start to develop, do they not, they kind of put some layers of… (laughs) reserves down and stretch it as they, as they grow. (Joshua's mother; child's BMI category: overweight)

She's saying ‘oh I'm fat, I'm fat, I'm fat’. And I'm saying but you're not fat. She tends to go sort of plump like, she's not fat, really fat. And then she shoots up again and she's skinny and then she stops and then her weight like, just her weight and then her height and then the weight. And then height again. (Amanda's mother; child's BMI category: overweight)

There were many parents who had teenagers classified by their BMI as overweight/obese and who considered their child's weight to be a concern. These parents described their child as needing to lose weight by changing their eating habits or levels of physical activity. Some expressed the view that their teenager's weight was verging on becoming a more major concern and that any further weight gain would be a ‘tipping point’ for taking action. For many of these parents, however, their concerns were not a straightforward acknowledgement of their child being overweight or obese, as they situated their views within the context described above. Specifically, parents talked about how their teenage child was growing and their body shape changing, making it difficult for them to discern whether concern or action was justified:

I would like Caroline to, she's a very heavy, as she goes about she's very heavy and..not lethargic but almost, y'know, I'd like to see her sprout about more and a bit more life about her body and … I think it could just be due to her weight. (Caroline's mother; child's BMI category: overweight)

I know that she can afford to lose a couple of pounds and she has come down a lot but … she's chunky… she was size 18 when we went on holiday … two summers ago … last year, she was about a 16 and today she's 12 to 14 which is good but as I says, she's chunky. (Nicole's mother; child's BMI category: obese)

Across all these categories of parental views, genetic explanations were very often put forward as a way of making sense of children's weight or body size. Boys and girls across the BMI range were seen as ‘taking after’ family members, as has also been observed in another Scotland‐based study.^16^ In our study, this included having similar body shapes to others or confronting genetic challenges to gain/lose weight, either now or in the future. The following comments are typical across the sample:

We're short legged, stocky type, that is our breed, both families, both myself, my family and my husband's and Finlay's got quite short stocky legs, he's a good rugby player size and he would like to be longer and leaner but to a certain extent I don't think that's his make‐up. (Finlay's mother; child's BMI category: overweight)

I mean she's a big lassie but she's not, she's not fat. All‐all of her dad's side is all big, eh… I says to her, if you were meant to be thin, you'd be thin. (Jodie's mother; child's BMI category: obese)

## Parental attitudes towards and experiences of addressing teenagers' weight

Parents described multiple strategies for trying to help manage their teenagers' weight, which were grounded within the social, emotional and biological context of ‘being a teenager’. Many parents talked about trying to find a balance between dealing with puberty and the ‘the teenage years’ and wanting to guide their child towards managing their weight in an appropriate manner. Younger children were perceived as ‘getting away with’ weight issues, both in terms of being less likely than teenagers to experience weight gain or excess weight and also in terms of parents, not children themselves, being responsible for any perceived action needed to counter this. Teenagers, by contrast, were viewed as making the transition to becoming more autonomous and therefore needing to take greater responsibility for their own actions.^5^, ^9^ In this sense, the teenage years were seen as the first time when parents had to confront whether, and how, to guide their children on issues connected with weight and body size. In many cases, this was not considered an easy task.

Some parents reported that they tried to help their child to make healthier food choices to manage the impact of diet on weight. This included buying more fruit and fewer high fat/sugar snacks and drinks within the overall family food shopping or setting rules to limit teenagers' consumption of snacks such as sweets and confectionary. Measures like these were seen as an intrinsic part of parenting children and teenagers. Though parents frequently reported trying to advise their child how to eat more healthily, to be more active and to ‘watch their weight’, there was quite often a sense of exasperation expressed by parents about this approach; a perception was expressed they could only do ‘so much’, then it was up to their child to follow the advice, as the following examples illustrate:

I say ‘well, don't eat chocolate bars…You know, I don't buy them, I'm not encouraging you to do it so… ’(Judith's mother; child's BMI category: overweight)

He desperately wants to lose weight but he doesn't want to do anything about it, he wants me to do everything for him, which is not feasible because when he goes to the school he is just eating everything anyway… so he really has to want to do it for himself really, rather than anything else. (Nick's mother; child's BMI category: obese)

General weight management advice was usually tempered through parents' fears about giving teenagers a ‘problem’ (Caroline's mother; child's BMI category: overweight) or ‘turning her anorexic’ (Shona's mother; child's BMI category: overweight), and these perceptions strongly influenced parental strategies for addressing their child's weight management. Concerns were often informed by parents’ own past and present experiences of weight management, as discussed earlier in the paper. Elspeth's mother's comments are typical in this respect:

When she did start to put on a bit of weight, I think just as every child has a tendency to do at the start of their teenage years and yes but it's a very… it's a very difficult line to tread because you do not want to push them towards anorexia so you can encourage them to eat healthily and all the rest of it but I am not wanting to put in her head that anything is particularly bad for her or she shouldn't eat …because I believe anything in moderation… and I've had… I've got issues with what I eat but I'm not prepared to start that with Elspeth so I do it in a very, very low key fashion. (Elspeth's mother; child's BMI category: normal)

Most parents reported that they would not condone their child starting a weight loss diet, whatever their perceived size. Diets, particularly low calorie ‘crash’ diets were viewed, overwhelmingly, as inappropriate for children and teenagers; hence, parents who were dieting themselves often said that they hid this from their children as it sent out the ‘wrong message’ (Alexander's mother; child's BMI category: overweight). Jodie's mother commented that ‘It's alright for me to do that, but not them’ (child's BMI category: obese).

Despite parental concerns about children dieting and developing psychological issues related to their weight, a few parents said they were quite blunt in their advice to their teenagers. These parents expressed exasperation that their child was having difficulty managing their weight and that they might be entering a trajectory which would mean on‐going weight management:

So what kind of things would you talk to her about?

Eh, ‘stop eating chocolate and stop eating cream buns, you know what you're doing to your body, you know what you're doing to yourself.’ She's concerned about her size. Em, Judith wants to be a dancer, you know, and there's not many dancers at five foot eight that have Judith's shape. You know, you just say to her ‘Judith, you keep eating the chocolate’ cos she'll say ‘oh my tummy feels really big’ or ‘my legs feel really big’ and you'll say ‘well, you know, you exercise a lot so obviously it's not through lack of exercise that you are the shape you are, you know, start looking at your diet Judith, start eating a bit more vegetables, start eating more variety, you know, stop eating bread!’ (Judith's mother; child's BMI category: overweight)

## Discussion

These findings from interviews with parents suggest that they have firmly held attitudes and views about their teenage child's weight and the efficaciousness and appropriateness of weight management at this point in the life course. The views expressed were often grounded in parents' own experiences of weight issues; lay notions of heredity; a lack of knowledge and understanding of physiological changes which might be ‘normal’ during puberty; and, a desire to protect their teenager's health and emotional wellbeing.

Across socio‐economic groups, the majority of parents expressed concerns about their own weight and reported difficulties with their own weight management, and this appeared to influence how they subsequently approached and viewed the management of their child's weight. Parents also shared their concerns about engendering anxiety in their child, including fears of triggering an eating disorder, which, as some of the comments indicated, may have arisen from their own negative experiences of dieting. These findings, and those from other studies,^17^, ^18^ suggest that the stress and stigma of a lifetime of being overweight and the impact this has on later, parental, weight management strategies needs to be more fully considered when planning services for teenage obesity management that rely on parental engagement.^19^

However, there might be other, underlying reasons which could be driving how parents view and manage overweight or obesity during adolescence. Parents do not exist in a social vacuum, and their views and approaches to parenting are shaped through their interactions with others who are, often, ‘like them’.^20^ Similarly, obesity is experienced within a particular socio‐cultural environment; hence, it is pertinent to consider the influence of socially‐constructed attitudes about raising a child in a so‐called obesogenic society and whether this might lead parents to ‘read’ their children's bodies in a particular way.^21^ In this study, we found no discernible difference in the views of parents from higher or lower SES groups; therefore, it is possible that similar degrees of normalization regarding overweight and ‘bigger bodies’ might have occurred across the socio‐economic spectrum.

Parents in this study often struggled to find appropriate words to define or interpret their child's weight, with use of phrases like ‘he has a tummy’ being put forward to describe a teenager's physical size or shape whilst not passing judgement about whether the teenager was ‘too big’. As reported in earlier UK studies,^22^ parents often do not consider their child's weight to be unduly problematic. Our findings suggest that even when parents are concerned about their child's weight, they often normalized their child's body size as being within an acceptable range. One explanation could be that individuals often cope with the reality they see by interpreting it against their own terms of reference,^23^ rather than through using the terms of reference that might be imposed by health professionals. Hence, attributing weight gain to the ‘normal’ changes associated with puberty, for example, or deciding that a teenager is not ‘too big’, might allow parents to quieten any anxiety they might feel about their son or daughter being or becoming overweight. This might allow parents to bypass the perceived moral judgements often associated with not achieving optimal health or body size.^24^ The social value of avoiding obesity is such that it is perhaps not surprising that parents locate their children on the ‘acceptable’ part of the health/weight spectrum, even if this seems to go against the evidence before them.

In addition, the normalization of a child's body size, whatever their BMI, means individuals can define themselves as ‘good’ or responsible parents.^25^ Downplaying the extent of a child's weight gain or explaining overweight as being down to genetics or bad luck, as many parents in this study did, may help to maintain a parent's position as being ‘better than’ a parent who ‘allows’ a child to become overweight or obese.^26^ A parent who is willing to label their child as being obese might need to re‐define the type of parent they are as a result; as Southwell and Fox argue, this could ‘open the psychological door’ (Southwell & Fox, 2011: 628) in terms of the implications that might then arise.^26^ These authors also found that parents with mental or physical problems (when symptoms might include being overweight) may feel particularly vulnerable to signs that they are a ‘bad’ parent.^26^ Seen in this light, perceiving of one's child as having a normal body size, even when faced with disconfirming evidence, might be a reasonable stance to take.

Just as defining a child as obese or ‘normal’ is socially inscribed, so too is help‐seeking behaviour;^27^ therefore, it is perhaps not surprising that parents did not seem to easily seek help for teenage weight gain or obesity, even when concerns about a child's weight were voiced. Unless a particular threshold is crossed or a crisis‐point arrived at (which a minority of parents in this study seemed to acknowledge they had almost reached) most parents might stay in what Biddle *et al*. describe as a cycle of avoidance.^27^

Parents’ worries about weight‐related discussions triggering an eating disorder, which this and other studies have found,^26^, ^28^ might be explained in terms of parents preferring not to directly confront the morally‐loaded issue of their child failing to maintain a socially acceptable weight. The findings show that parents often skirted around the issue of weight when advising teenagers. ‘Discussing vs. telling’ has, however, been put forward as a positive strategy for parent–teenager communication about weight.^28^ In addition, most studies drawing on social theory advocate tackling the underlying context of health rather than confronting an individual him/herself;^29^, ^30^, ^31^ therefore, helping parents to find ways to create a healthy home in which to support teenagers, rather than designing interventions whereby parents more directly tackle weight within the family, could be worthwhile.^28^ The findings show that parents were comfortable restricting the types and amounts of some foods available in the home; therefore, such strategies could be advocated without directly addressing weight with teenagers. If, as our data suggest, the teenage years are viewed by parents as a time when children need to take some responsibility for their own weight management, however, then this might also indicate the need for interventions to further and, perhaps more imaginatively, engage young people in partnership with their parents. Young people certainly need to be consulted about the best ways to facilitate this.

Health educators and service providers do not currently have the answer to addressing obesity and prevalence rates, which, therefore, continue to be a concern in countries like the UK. We suggest that it is vitally important that health‐care providers understand the origins of parental attitudes to weight and acknowledge that these drive and regulate norms and what parents perceive as appropriate weight management strategies for their teenage children. This could help in developing and sustaining patient‐centred care and services.

## Conflict of interests

There are no known conflict of interests relating to this research and the authors.

## Funding

The research drawn on was funded by the University of Edinburgh; NHS Health Scotland; and the UK Economic and Social Research Council (Ref: RES000231504)
